# Stacked Ensemble Model With Explainable AI for Early Detection of Heart Disease

**DOI:** 10.1002/ansa.70072

**Published:** 2026-03-03

**Authors:** Nazmun Nahar, Sanjatul Hasan Siam, Joy Bhowmik, Ayesha Nasrin Ripa, Md. Hasan Imam, Haw Jiunn Woo, Hamid Osman, Mayeen Uddin Khandaker, Shams Forruque Ahmed

**Affiliations:** ^1^ Institute of Information Technology Noakhali Science and Technology University Noakhali Bangladesh; ^2^ Centre for Ionics University of Malaya Department of Physics Faculty of Science Universiti Malaya Kuala Lumpur Malaysia; ^3^ Department of Radiological Sciences College of Applied Medical Sciences Taif University Taif Saudi Arabia; ^4^ Applied Physics and Radiation Technologies Group CCDCU Faculty of Engineering and Technology Sunway University Bandar Sunway Selangor Malaysia; ^5^ Faculty of Graduate Studies Daffodil International University Daffodil Smart City, Birulia Savar Dhaka Bangladesh; ^6^ Department of Physics College of Science Korea University Seoul Republic of Korea; ^7^ School of Mathematical Sciences Sunway University Bandar Sunway Selangor Darul Ehsan Malaysia; ^8^ Miyan Research Institute International University of Business Agriculture and Technology Dhaka Bangladesh

**Keywords:** ensemble model, explainability, explainable artificial intelligence, heart disease, machine learning

## Abstract

Heart disease (HD) is still one of the most common causes of death around the world. Early detection is very important, but it is often hard to do because the symptoms are not specific and the models are not very clear. We propose a two‐layer stacked ensemble that combines four base learners—Support Vector Machine, K‐Nearest Neighbors, Naïve Bayes, and Decision Tree—with Logistic Regression as a meta‐learner. This ensemble was trained on a balanced sample from five public HD datasets. The model outperforms standard baselines with 93.69% accuracy, 93% F1‐score, and 94.7% AUC. To ensure that predictions are clinically interpretable, we offer global explainability through SHAP summaries, feature importance, and stacked plots, as well as ELi5 permutation–importance plots. Both identify ST slope and chest pain type as the most significant predictors, consistent with clinical understanding. We use LIME, ELi5 text explanations, SHAP force plots, and waterfall plots to illustrate how different features of a person affect their risk. We utilize SHAP interaction values to examine feature interactions and confirm nonlinear relationships using 1D and 2D partial‐dependence plots. Finally, we generate counterfactual explanations to demonstrate minor, plausible changes based on domain knowledge that would affect a prediction. This is useful both for auditability and for conversations around patient‐centred care. The framework combines global and local explainable artificial intelligence with strong predictive power to improve decision support and accuracy in HD risk assessment.

## 1 Introduction

Heart disease (HD) is a prevalent and deadly disease that mainly affects middle‐aged and elderly individuals and has a higher risk in men as compared to women [1]. In Bangladesh, non‐communicable disorders (NCDs) represent 67% of all mortality, especially cardiovascular disease, which alone is responsible for around 30% of deaths [2, 3]. Globally, HDs claim the lives of one‐third of the population [4]. The populations affected from low‐ and middle‐income nations, such as Bangladesh, are responsible for 80% of these deaths [5]. HD kills approximately 17 million people a year around the world, and it is particularly common in Asia [2, 6, 7]. Risk factors for HD include age, gender, smoking, family medical history, cholesterol levels, eating habits, hypertension, being overweight, lack of exercise, and alcohol use. Some HD can also be due to inherited factors, such as high blood pressure and type 2 diabetes. Some risk factors can be mitigated. Apart from these characteristics, lifestyle behaviours, including but not limited to diet, physical inactivity, and obesity, are also well‐established risk factors [8, 9, 10]. Different types of HD include ischemic HD, angina, cardiomyopathy, chronic heart failure (HF), congenital HD, cardiac arrhythmia, and myocarditis. Given the risk conditions, the probability of HD development is not easily calculated manually.

HD is diagnosed through a clinical assessment, investigation of risk factors, and medical imaging. Yet they cannot reliably forecast whether HD will occur, especially during the pre‐symptomatic stages in which disease signs are absent. As healthcare data become increasingly accessible and abundant, many researchers have started using machine learning (ML) methods in this problem domain to achieve better diagnostic accuracy. ML algorithms operate in a methodical manner to predict the likelihood of HD by identifying patterns in massive datasets, thereby making them more efficient than traditional methods. Despite the development of ever more sophisticated diagnostic tools and medical imaging, these classical forms of diagnosis have failed to predict HD at an early stage, where intervention could be most effective. This has brought interest towards the use of ML models that can be employed to identify and analyse large healthcare‐aiding datasets to predict the potential risk of disease based on patterns discovered within [11]. Due to their excellent predictive accuracy, many ML models, such as Support Vector Machines (SVMs), Artificial Neural Networks (ANNs), and Ensemble Learning (EL) models, are purely empirical; however, some of these models have been labelled as ‘black box’ approaches. Without a smooth way of assessing how a prediction is made, models become difficult to clinically adopt and utilize, as clinicians are unable to trust such models, whilst not knowing how a prediction was derived. EL is one of the major techniques to improve the performance of ML models, which uses many ML models to predict its output. These algorithms have excellent performance because of their algorithmic competency, yet they are considered black‐boxes, and their structure of perception is difficult for healthcare professionals to fully comprehend.

This study is motivated to help bridge the research gap between model interpretability and prediction accuracy for HD detection. Prior work has largely focused on the enhanced predictive accuracy of ML models for HD, often trading off interpretability and feasibility in real‐world clinical settings. These black‐box models have greatly improved prediction performance; however, we have little understanding of how to build models that are similarly accurate and interpretable. Their predictive models need to be accurate, but they also need to explain the processes involved in an interpretable way to enable sound decision making. Thus, the knowledge gap our paper fills is interpretable ML for HD prediction. These models are either accurate but not interpretable or interpretable. To overcome the limitations of black‐box models, this work proposes an explainable and transparent ensemble framework, Stacking ML‐XAI. This stacking ensemble framework is a combination of various ML algorithms like K‐Nearest Neighbors (KNN), SVM, NB, and DT to increase the accuracy of prediction. Simultaneously, it employs XAI methods, ensuring the explainability and interpretability of the model's decisions.

The study's key contributions are summarized as follows:

- For the prediction of HD, a stacking ensemble method has been developed.
- To make the model more trustworthy and to achieve a good balance between accuracy and interpretability, which would be easy for clinicians or physicians to understand and use, the model's internal explainability has been included.
- With the use of SHAP plots, doctors may see how each characteristic contributes to the overall decision‐making procedure and which features are the most important when generating disease predictions.
- SHAP force plots, SHAP waterfall plots, and LIME explanations, providing patient‐level justification of predictions.
- SHAP interaction values and 1D/2D partial dependence plots (PDP) show that features interact nonlinearly.
- The model provides counterfactuals where the minimum set of feature changes can change a diagnosis.

## 2 Related Works

Recent advancements in ML research have prompted increased usage of ML algorithms for HD prediction and risk monitoring. Various strategies have been employed with different kinds of datasets, achieving performance compatibility and validating their performance in various algorithms such as SVM, KNN, and EL [12, 13]. The summary of studies on ML models and their performance metrics for HD prediction is mentioned in this section.

### 2.1 Studies on Specific ML Algorithms

#### 2.1.1 Support Vector Machine

SVMs have frequently been employed in HD prediction due to their strong classification performance. For example, Chowdhury et al. [14] established a questionnaire and collected data from several local hospitals and healthcare institutions in the Sylhet district of Bangladesh. Their dataset has 18 attributes and 564 instances. With a 91% accuracy rate, SVM was remarkably accurate. However, despite its strong predictive power, the model lacked interpretability, an essential requirement for clinical adoption. Without transparent reasoning behind predictions, clinicians may be reluctant to rely on such systems in real‐world decision‐making.

Liu et al. [15] proposed and developed multiple ML techniques and feature selection approaches for building an accurate stacking model fusion for a CVD classifier, but with high dimensional data. The researchers used a fused heart dataset from various UCI ML libraries, along with one other publicly available Heart Attack dataset, to train and validate their model. They achieved an accuracy of 89.86% and a 91.35% F1‐score using the SVM component of a stacking fusion model on the UCI Heart Disease dataset. Using the SHAP method, they highlighted the most significant components contributing to the prediction of CVD. The smaller size of the sample, the absence of external validation, and the explainability of the stacking model were some of the limitations of the study, the authors acknowledged.

#### 2.1.2 K‐Nearest Neighbors and Random Forest

In some studies, the usage of KNN and RF algorithms, as single models or in combination with other techniques, for HD prediction has been examined. Assegie et al. [16] used the KNN classifier to train an HD prediction model with a Kaggle dataset containing 1025 observations. With an accuracy of 91.99% reported in the study, KNN shows an effective performance in HD prediction. It studies the influence of various *K*‐values, reporting an optimum *K* = 1 performance. Dataset features include age, chest pain type, and cholesterol, with the relationship between features determined by Pearson's correlation. The limitations of this study, nevertheless, include the use of only KNN and the absence of comparisons to other state‐of‐the‐art algorithms.

Pal et al. [17] used RF to perform HD prediction on 14 clinical features from a set of 303 samples. The model achieved an accuracy of 86.9% with 90.6% sensitivity, 82.7% specificity, and a high area under the curve to differentiate cases with HD. HD was also shown to be predictable with RF. Because RF is highly accurate and has a black‐box output, it is difficult to know which features are important. The black‐box nature of RF provides poor interpretability about how features contribute to predictions, which complicates clinical interpretation and reduces trust on the clinical side of healthcare settings.

Kumar et al. [18] proposed a framework for predicting HD using an enhanced KNN, called E‐KNN. E‐KNN performed with an accuracy of 90.10% after applying the chi‐square test to select the features. The approach was compared with traditional methods, including KNN, SVM, and CART. Furthermore, the E‐KNN performed better in recall, precision, and F1‐score. The chi‐square test further improved the model of feature reduction from 13 to 11. However, these techniques are limited by their non‐explainable artificial intelligence (XAI) properties. While the developed model performs well, the black‐box prevents clinicians from understanding its decision‐making process, which makes it difficult to trust and put in production for real‐world medical practices.

### 2.2 Ensemble Models and Hybrid Models

Ensemble model (EM), especially stacking methods, have been widely explored for HD prediction.

Kavitha et al. [19] proposed an ML‐based HD prediction model but utilized the combined method of DT and RF algorithms. It is a binary classification problem designed for populations with HD from the Cleveland database, involving 303 instances. The hybrid model, which combines these, outperformed the individual algorithms, achieving an accuracy of 88%. In comparison, RF only reached 81%, and DT, at its best, around 79%. Users can input medical data and get predictions using a simple graphical interface. On the other hand, some limitations of this study include its relatively small dataset size, which can affect generalizability, and its inability to compare with more advanced models such as SVMs or ANNs.

Cenitta et al. [20] presented a Hybrid Residual Attention‐Enhanced LSTM (HRAE‐LSTM) model aimed at enhancing the diagnosis and assessment of ischaemic heart disease (IHD) employing the UCI Heart Disease dataset. By fusing residual attention mechanisms with LSTM networks, the model effectively captures temporal and complex feature dependencies and thus obtains a high accuracy (97.71%). The data pre‐processed using the fuzzy‐based multiple imputation method makes predictions stable and reliable due to the imputed data having a more accurate predictor. Even though the model performs well, it lacks XAI, which makes it difficult for the physician to interpret the decision‐making process of the model. This is a key aspect for clinical trust and usability.

In their study, Adhikari et al. [21] used ML models, such as EM, that combine different classifiers, to predict potential heart risks in patients. The paper proposed and developed a module to learn prediction for patients based on an individual feature set. They also used an ensemble approach to build two models using voting and averaging methods, and evaluated their performance based on the individual model results. Their EM was 88% accurate. It does not provide the rationale for the better performance of the EMs and does not perform feature importance or model interpretability in a clinically relevant manner.

Abdollahi et al. [22] proposed a hybrid method for feature selection and EL methods for HD identification. Furthermore, the study also evaluated HD datasets using different feature selection techniques and ML algorithms. The proposed technique attained an accuracy rate of 97.57% on the HD dataset, surpassing previous achievements. There was no clear explanation provided by the authors on how the genetic algorithm operated or how it selected the ideal feature subset. In addition, they did not compare their hybrid method to other standard ensemble methods such as bagging, boosting, or stacking, limiting the potential to contextualize their findings within the wider context of ensemble‐based diagnostic models.

Noor et al. [23] proposed a new stacking model called PaRSEL, which was specifically designed for HD diagnosis. It also emphasizes the importance of reduction of data dimensionality and balancing the data to handle problems in the dataset, such as imbalance. To address these challenges, eight different methods, including ProWRAS, LoRAS, LDA, RFE and FA, are implemented. Then, SHAP values are also incorporated to explain model predictions at the level of each feature. Results from the study yielded an accuracy of 93.69%, an F1‐score of 93% and an area under the curve (AUC) of 0.947. The limitation of the study is that complex models like PaRSEL are designed to predict HD face issues like overfitting and high computing cost due to enormous and intricate datasets, and do not offer any local interpretability of the model.

Abdulsalamal et al. [24] investigated the performance of Quantum ML (QML) to predict the HD risk by designing an EL model based on QML algorithms. The study used several pre‐processing methods and the Cleveland dataset. Bagging‐QSVC model provided the best accuracy, at 90.16%. The study also shows the importance of each feature and its contribution to the prediction, using SHAP plots. The study had some limitations, for example, using a simulator instead of a real quantum computer and utilizing a small and biased dataset. Furthermore, the paper did not discuss the scalability and robustness concerns of QML algorithms.

### 2.3 Explainability and Local Interpretability

Although ML models have shown great potential for predicting health outcomes, frequently reaching high accuracy by learning complex patterns from training datasets, the black‐box nature of these models creates substantial barriers to their clinical use. The black‐box characteristic of many ML algorithms means that it is challenging for healthcare delivery professionals to understand the rationale behind a given prediction, breaching their trust and limiting its adoption in practice. Thus, the incorporation of XAI into HD prediction models is a crucial step towards this end. This discrepancy in performance can hamper the acceptance of predictive systems in clinical settings. Yet, XAI techniques can play a significant role in bridging this divide by improving model generalization on the one hand and clinical interpretability on the other, thereby supporting the credibility of predictive systems to be used as a part of routine medical practice [25].

A similar guidance from the World Health Organization (WHO) highlights explainability as a fundamental principle for the ethical use of AI in health systems [26]. Transparency is necessary to support clinical reasoning, and it complements a global initiative to deploy responsible and trustworthy AI in medicine. Zhou et al. [27] provided a global solution for the prediction of HF in patients with ischemic HD (IHD). This dataset included anonymized Hospital Discharge Records (HDRs) for patients in Sichuan Province in China during the years 1 January 2015 to 31 December 2019. This method involved creating three comorbidity networks (PDN, BDN, DSN) to portray disease patterns over time. New edge score, node score and rank‐based score were proposed to assess disease progression on a network basis. The DXLR EL model that incorporates all these variables besides classic demographic information achieves better precision, recall, accuracy, F1 score and AUC than six classical ML models. Feature importance was evaluated using the SHAP approach. There are limitations, including the retrospective methodology and non‐cancerous dataset specificity, calling for cautious interpretation.

Santhosh et al. [28] performed a comparative analysis of several ML models, namely Logistic Regression (LR), Random Forest (RF), SVM, KNN and Naïve Bayes (NB), for the prediction of HD utilizing the UCI Heart Disease dataset. The RF model had the best accuracy (about 90%–94%), which shows that it is very effective at making predictions for early diagnosis. The study identifies significant determinants, including chest pain type, cholesterol level and maximum heart rate.

Bairy et al. [29] proposed a hybrid RF and SVM model to increase the reliability of HD screening, based on the UCI Cleveland dataset. Using efficient pre‐processing and model fusion, the method achieved an accuracy of more than 95%. This outperformed other classical classifiers, such as Decision Tree (DT), KNN and LR. The hybrid model combines the strong feature selection ability of RF and the strong classification ability of SVM, making the prediction more robust and reliable.

Guleria et al. [30] aimed to build an XAI framework for CVD prediction based on several classification algorithms. This work used the CVD database with 303 instances and 14 features. They also employed techniques such as weight initialization from other features, normalization and feature selection for dimensionality reduction. Moreover, several optimization techniques were adopted to improve classifiers’ performance. SHAP was demonstrated in the study to provide fair and interpretable explanations of both the model predictions and feature importance. Age, sex and cholesterol were the most relevant features for HD prediction. Utilizes a small size and highly imbalanced dataset, which may potentially limit generalizability and the robustness of the models and explanations employed. Moreover, it did not conduct model comparisons over XAI‐based models for local interpretation, which may restrict generalization and add novelty to the framework proposed.

The literature reviewed shows that although high performance has been achieved for HD prediction, using various ML algorithms, the studies are limited with respect to model interpretability and local explainability. To address this gap, we propose a stacking ensemble model (SEM) that achieves both high performance and good interpretability by combining SHAP and LIME, rendering it efficient, transparent and optimal for use in the clinical setting.

## 3 Methodology

**FIGURE 1:**
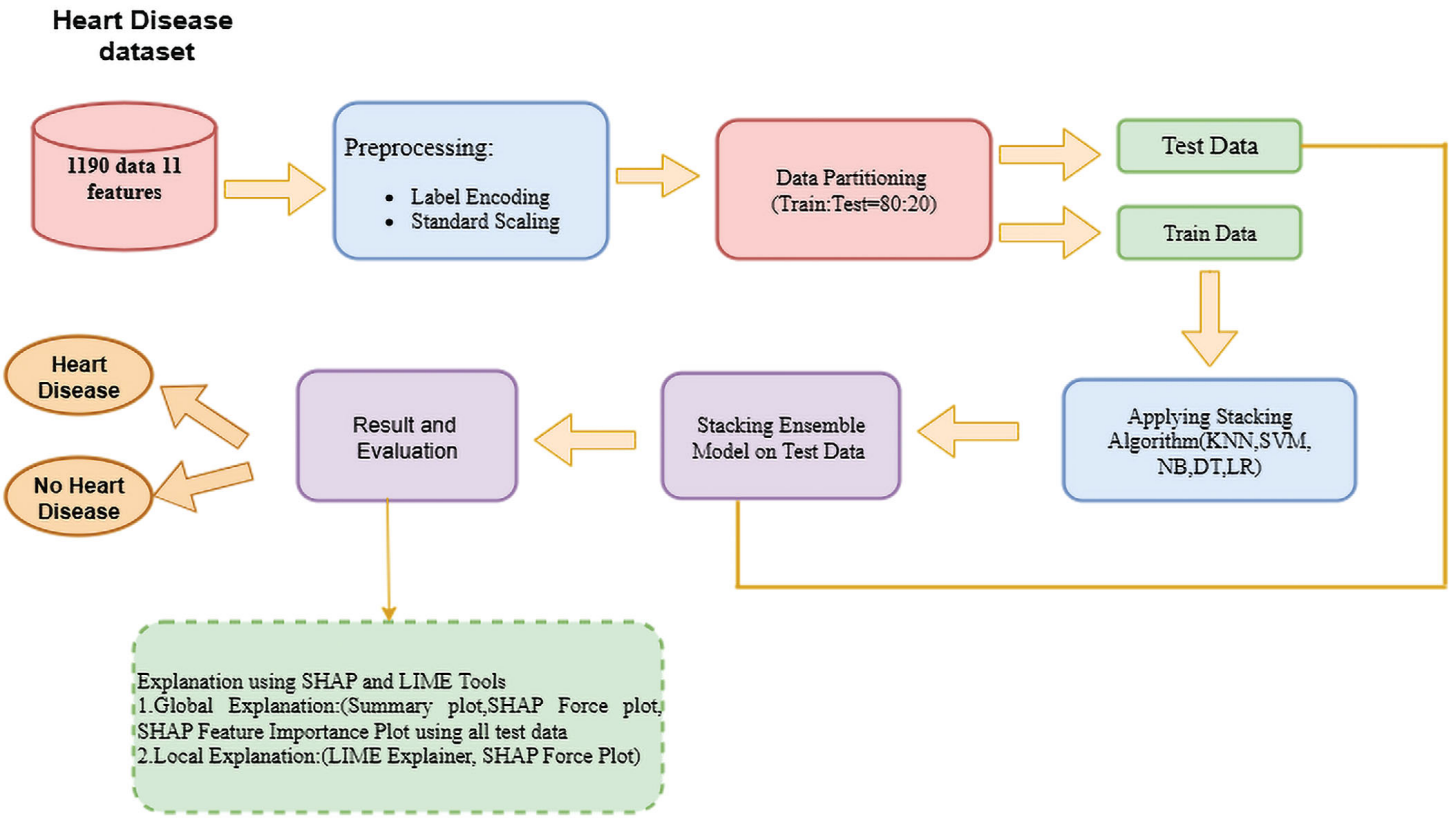
Overview of the proposed stacking ML‐XAI framework for HD prediction.

The overall approach to HD prediction is presented in Figure 1. It begins by fusing multiple datasets, like the Heart Dataset, which comprises the Cleveland, Long Beach, VA, Hungary, Switzerland and Stalog (Heart). From the five datasets, we extracted eleven useful features. Perform data cleaning and pre‐processing since there were missing values and the categorical data needed to be encoded to make them readable by ML models. The dataset was divided into training and testing groups in 80:20 ratio. Standardization method was also used to normalize the data to have a higher consistency and gain more uniformity among the models. The sections below explain each step in more detail.

### 3.1 Dataset

The heart dataset contains five different datasets, which are the Hungarian, Cleveland, Long Beach, VA, Switzerland and Stalog (Heart) data, taken from Kaggle [31]. Out of 1190 samples, 272 duplicates were eliminated, resulting in 918 unique sample datasets. Eleven significant features were picked from five datasets to create the resulting Heart Dataset, as seen in Table 1. Table 1 provides feature descriptions, whereas Table 2 shows statistical values for the dataset. This dataset was chosen because of its widespread availability for predicting HD, making it a popular choice among academics for model development. Hence, it would be advantageous to evaluate the suggested model in comparison to others and identify areas for improvement.

**TABLE 1:** Comprehensive description of features in the heart dataset.

ID	Feature name	Description	Possible values/units
1	Age	Age of patients	Year
2	Gender	Patient's gender	Female, male
3	Chest pain type	Type of chest pain	TA (typical angina), NAP (non‐anginal pain), AA (atypical angina), AS (asymptomatic)
4	Resting BP S	Resting blood pressure	mm Hg
5	Cholesterol	Serum cholesterol of the patients	mg/dL
6	Fasting blood sugar	Fasting blood sugar value	False, true
7	Resting ECG	Showing the resting electrocardiogram	Normal, having an ST‐T wave abnormality, showing probable or definite left ventricular hypertrophy
8	Max heart rate	The highest heart rate value	Heart rate from 71–202
9	Exercise angina	Whether individuals experience exercise‐induced angina or not	Yes No
10	Oldpeak	ST‐segment after exercise	ST value
11	ST slope	The ST section's slope at the movement's peak	Upsloping, flat, downsloping
12	Target	Class value	Heart disease, normal

**TABLE 2:** Summary of the heart dataset.

	Age	Sex	Chest pain type	Resting bps	Cholesterol	Fasting blood sugar	Resting ECG	Max heart rate	Exercise angina	Old peak	ST slope	Target
Count	1190	1190	1190	1190	1190	1190	1190	1190	1190	1190	1190	1190
Mean	53.72	0.76	3.232	132.15	210.36	0.21	0.69	139.73	0.38	0.92	1.62	0.52
Std.	9.358	0.42	0.935	18.36	101.42	0.40	0.87	25.51	0.48	1.08	0.61	0.49
Min	28.00	0.00	1.000	0.00	0.00	0.00	0.00	60.00	0.00	−2.60	0.00	0.00
25%	47.00	1.00	3.000	120.00	188.00	0.00	0.00	121.00	0.00	0.00	1.00	0.00
50%	54.00	1.00	4.000	130.00	229.00	0.00	0.00	140.00	0.00	0.60	2.00	1.00
75%	60.00	1.00	4.000	140.00	269.75	0.00	2.00	160.00	1.00	1.60	2.00	1.00
Max	77.00	1.00	4.000	200.00	603.00	1.00	2.00	202.00	1.00	6.20	3.00	1.00

**FIGURE 2:**
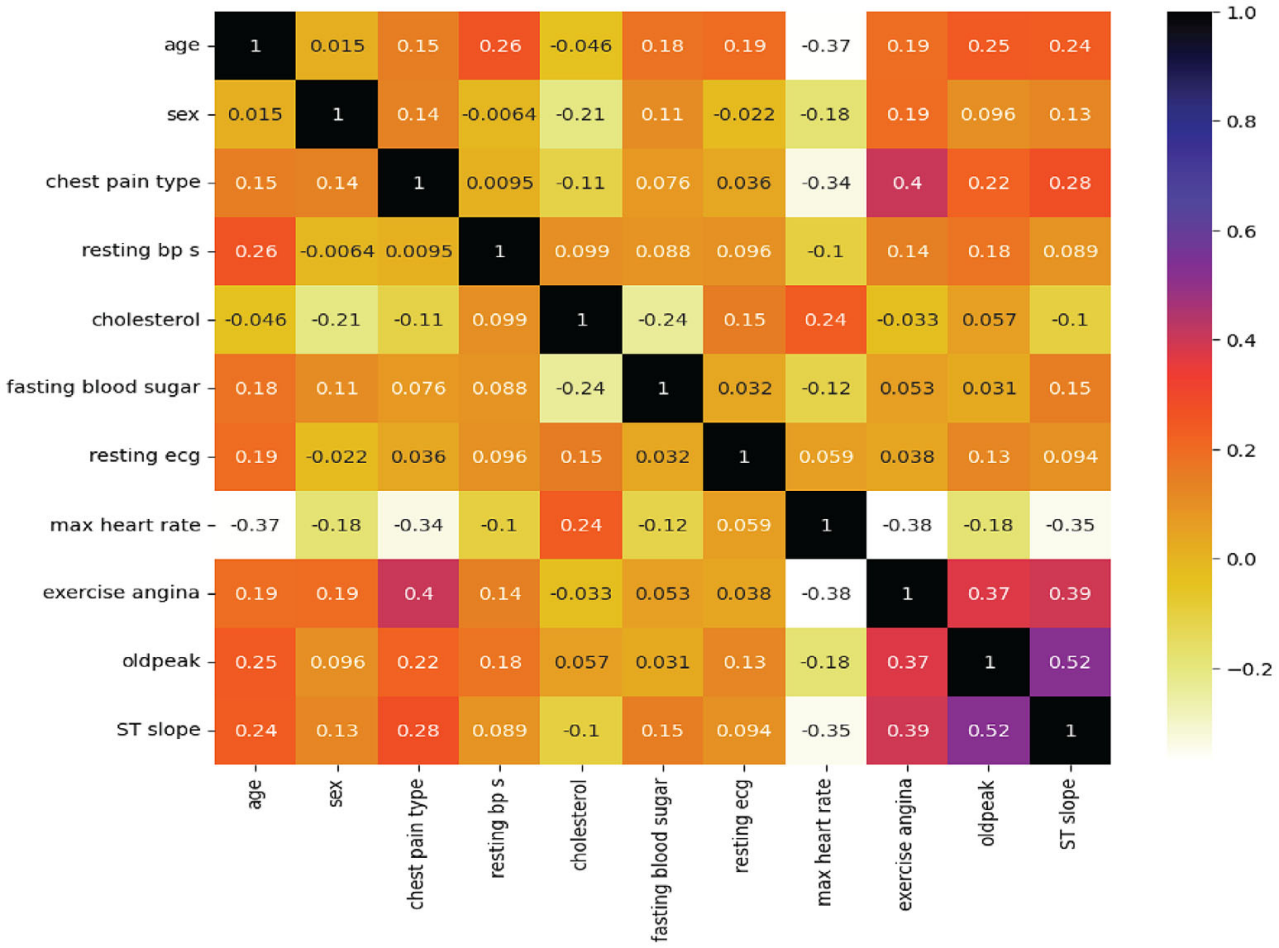
Pearson correlation map of heart dataset.

Figure 2 presents the Pearson correlation matrix for the selected features in the dataset, providing insight into the strength as well as the direction of relationships (linear) between paired variables. It computes the linear relationship between the two features based on their respective values. The correlation coefficients' values ranged from −1 to +1. As the value approaches 0, the correlation decreases. There is no relationship when the value is 0. A value nearer 1 or −1, respectively, indicates a stronger positive or negative correlation. The st_slope and oldpeak showed the most positive association. Age versus sex and resting ECG versus workout angina, also positively correlated. The features don't significantly negatively correlate with one another.

### 3.2 Data Pre‐Processing

Before developing the predictive model using a stacking ensemble approach, the HD datasets underwent a thorough pre‐processing phase. These datasets, extensively utilized in cardiovascular studies, have undergone extensive curation and are pre‐cleaned, which increases accessibility for researchers and reduces the preliminary effort required during data preparation. The wide documentation and numerous mentions in academic studies show their reliability. The pre‐processing went through a few steps, starting with detecting and fixing missing or duplicate rows. Missing values, including measurement errors, incomplete records or data loss, can have a severe impact on both the statistical validity of the model and its interpretability. Instead of excluding incomplete observations that might reduce the representativity of the dataset and bias the results, this study solved the problem of missing values ​​using imputation strategies. This means that missing entries were filled with a constant specified by the user or the mean value of the feature itself, based on whether the variable was categorical or continuous, and how it was distributed. This approach ensures data consistency and enables better model performance.

Feature scaling was another vital part of the pre‐processing, as it attempts to ensure that each input variable contributes equally to the prediction process of the model. Normalization and standardization were assessed as two often used methods, and standardization was used as the final method. Standardization is done by centring a feature, which means subtracting the mean, and then scaling it by dividing by the standard deviation. This transformation, which is mathematically expressed in Equations (1)–(3) reduce the impact of outliers while improving the convergence and stability of the learning algorithms. Standardization resolves this issue by scaling numerical features to the same range, making the algorithm more efficient and decreasing the potential damage of one feature dominating over others in the model output.

(1) $$\;x^{\prime }=\frac{x-\mu }{\sigma }\;$$

In Equation (1), *x*′ is a normalized value of a data point. It is derived by taking *x*, subtracting the mean (*μ*) from it, and then dividing the difference by the standard deviation (*σ*).

(2) $$\mu =\frac{\sum_{i=1}^{N}x}{N}\;$$

Equation (2) represents the mean (*μ*) as the summation of all data points (*x*_i_) and dividing it with total data points (*N*).

(3) $$\sigma =\sqrt{\frac{1}{N}\sum_{i=0}^{N}{\left(x_{i}-\mu \right)}^{2}}\;$$

Equation (3) gives the standard deviation (*σ*), which is the square root of the mean squared difference of each measurement from overall the sample mean. It tells how scattered our measurements are. Together, these equations take the raw data and convert it to a uniform format to ensure that features in different units or scales do not influence ML models.

For evaluating how well the model can generalize outside of the training data, the HD dataset was split into two subsets using the standard 80:20 ratio. Specifically, out of the dataset, 80% were designated for training, where the model would learn the statistics of the variables and the relationships between them. The other 20% was kept out as a testing set to independently assess how accurately the model can predict new instances. This is commonly used in ML and is done to make any performance metric (in this case, precision, but could also be accuracy, recall or AUC) as close as instances to reflect the model's actual ability to generalize, not its ability to recall specific instances from the training data. This procedure mitigates the risk of overfitting by ensuring that the training and evaluation phases are clearly separated. This contributes to a more accurate prediction of its expected performance on actual, real‐world use cases.

### 3.3 Model Development

The higher learning capacity of individual‐based learners in stacking and decreased correlation result in improved model prediction. Greater variability among individual learners leads to improved model fusion when the learners have already become more precise. This hypothesis is known as the ‘error‐ambiguity decomposition.’ Base learners should be chosen on the basis of the notion of ‘good but different,’ which involves evaluating individual learners' performance while also considering their dissimilarity. We prioritize accuracy by simplifying the model and choosing a stacking model consisting of a two‐layer structure with base learners and a meta‐learner. Thus, KNN, SVM, NB and DT were chosen as potential models for base learners to predict HD. We originally chose base learners with superior prediction performance based on accuracy. Ultimately, we picked three broadly representative models as candidates: KNN, SVM, NB and DT. The base layer of the stacked architecture typically acquires input features for the subsequent layer, and the model can simultaneously produce numerous characteristics.

**FIGURE 3:**
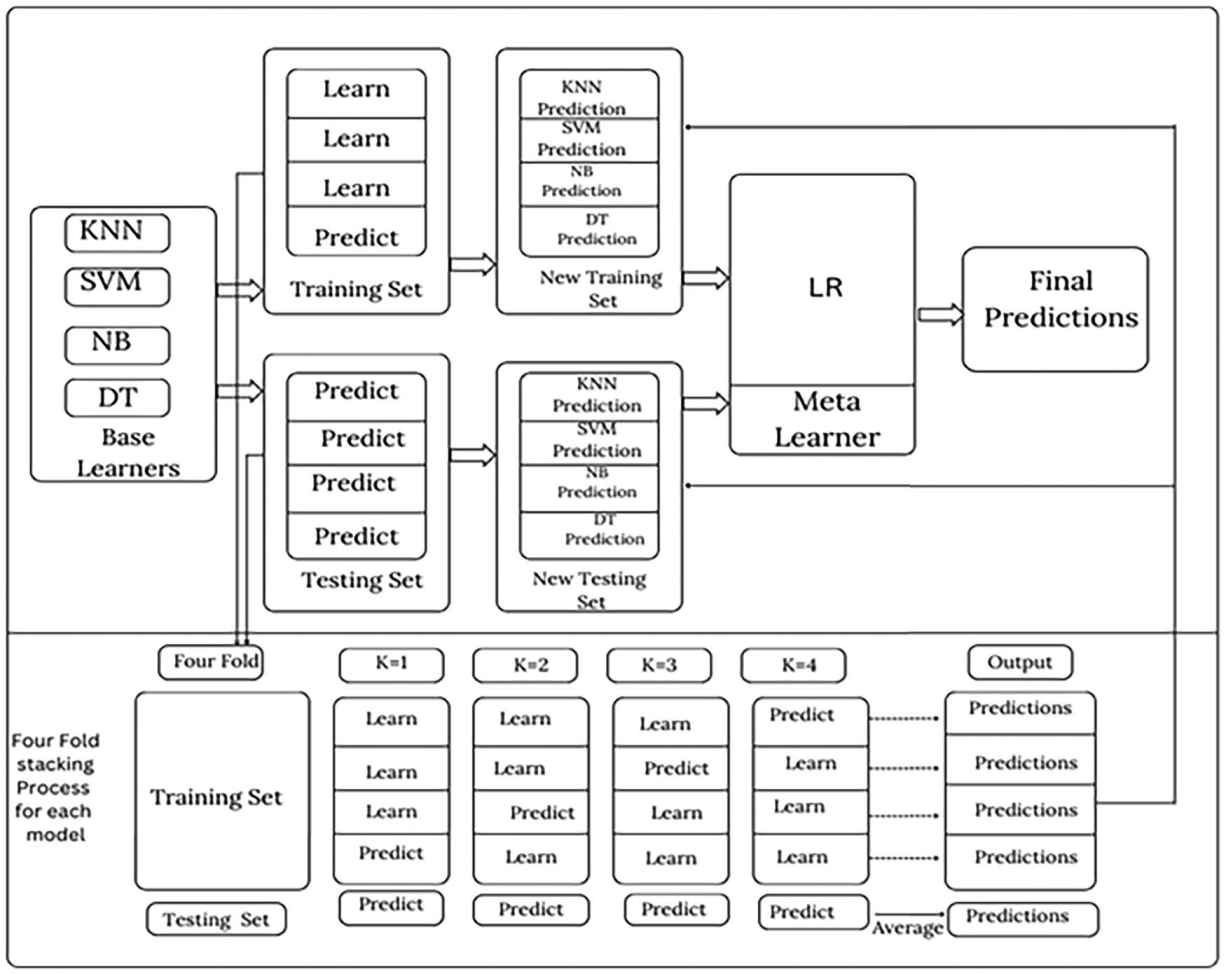
Architecture of the proposed stacking model.

Typically, the second layer of the stacked architecture consists of simpler classifiers such as generalized linear regression (GLR). Hence, for the prediction modality, we target LR to build our combined model. A complex, nonlinear transformation can be the base model for the initial layer of the stacked structure, meaning there is no need for an expensive transformation at the subsequent classifier selection layer. The architecture of the proposed stacking ensemble is shown in Figure 3, where the outputs of the base models are fed into the meta‐learner. The hierarchical framework allows the model to benefit from the use of different classification paradigms while preserving the computational efficiency and interpretability that are essential for its practical application in medical diagnostics.

Algorithm 1 **Stacking ensemble model (SEM) for HD prediction**

**Table:** 

	**Define**: A Training set: X and Testing Set: V. Base Learners b = {KNN, SVM, NB, DT}. bl is the base learner and a meta learner m = {LR}. X^b^ of bl is the training dataset for each base learner, and V^b^ of bl is the testing dataset for each base learner.
1.	For b = {KNN, SVM, NB, DT} do
2.	For k = 1,2…4 do
3.	X^b^→bl(use X^b^ to train bl)
4.	V^b^→bl →train_m_, V^b^→bl→test_m_ (predict V^b^, V^b^ by bl to get train_m_, test_m_)
5.	End for
6.	train_l_ = [train_1_+train_2_+train_3_+train_4_] (Vertical Stacking)
7.	test_l_ = [test_1_+test_2_+test_3_+test_4_]/4(Horizontal Averaging)
8.	End For
9.	train_new_ = [train_knn_, train_svm,_ train_NB,_train_DT_]
10.	test_new_ = [test_knn,_test_svm,_test_NB,_test_DT_]
11.	train_new_→LR_m_(use train_new_ to train LR_m_)
12.	test_new_ →LR_m_→Result_prediction_ (use LR_m_ to predict test_new_ to get the final result)
13.	Return Result_prediction_

The base learners comprise KNN, SVM, NB and DT. The training and testing sets are divided into the first steps using an 8:2 ratio. For each of the four base learners in the training set, we employ four‐fold cross‐validation (CV). One base learner generates four predictions, which are then stacked vertically onto a one‐dimensional matrix. The four basic learners may be aggregated into a four‐dimensional matrix to serve as the training input for the second step. In the testing set, we again applied the four‐fold CV model to make predictions about the initial testing set and acquire four more predictions. In this case, the outcomes are combined horizontally to generate a one‐dimensional matrix. This is done to guarantee that the same ratio would be maintained between the set that was used for training and the testing set. When it comes to the testing data for the second stage, it is possible to manipulate the same four base learners such that they merge into a four‐dimensional matrix. During the second stage, we prioritize these predictions and use LR in the meta‐learner to prevent overfitting. The results are then determined based on these predictions. Algorithm 1 explains the stacking model.

### 3.4 Time and Space Complexity of the Proposed Stacking Model

The SEM is a combination of multiple ML algorithms to enhance the precision and generalization power in predicting HD. As we know, ensemble methods offer better performance compared to a single classifier, but they also have more computational complexity. This complexity comes from training multiple base learners and using a meta‐learner for final classification. Thus, we study the computational complexity (time) and space complexity of the proposed model and the trade‐offs between the performances of stacked‐up models with respect to their computational requirements.

#### 3.4.1 Time Complexity

##### 3.4.1.1 Base Learners

The ensemble incorporates four base classifiers—KNN, SVM, NB and DT—selected for their diverse learning strategies and complementary strengths.

##### 3.4.1.2 K‐Nearest Neighbors

The time complexity for training is O(1) as there is no explicit training in KNN. However, for prediction, it has a time complexity of O(*n*⋅*d*), where n is the number of training samples and d is data dimensionality.

##### 3.4.1.3 Support Vector Machine

The training time complexity of SVM varies with the implementation. For non‐linear kernels (like the Radial Basis Function), this will be O(*n* ^2^⋅*d*), with *n* being the number of training instances, and *d* being the number of features.

##### 3.4.1.4 Naïve Bayes

The Time complexity of Naïve Bayes is O(*n*⋅*d*), with n as the number of samples and d as the number of features in each sample. It is a relatively simple model to compute.

##### 3.4.1.5 Decision Tree

The training complexity of DT is O(*n*⋅*d*), where *n* = number of samples, *d* = number of features.

#### 3.4.2 Meta‐Learner (LR)

##### 3.4.2.1 Logistic Regression

LR time complexity is O(*n*⋅*d*). Training and predicting are often done efficiently in this algorithm

##### 3.4.2.2 Complexity of the Stacking Model

It refers to the complexity of base learners and the meta‐learner. The model uses 4‐fold CV to train base learners, thus multiplying the computational cost of the base learner by four. Once the base learners have been trained, the predictions from these models are then passed to a meta‐learner (which adds another level of complexity). For example, because the model uses four base learners and a meta‐learner, some of these base learners (SVM with RBF and KNN) can be quite complex, as estimated by Equation.

$$O\left(4.\left(n^{2}.d+n.d\right)+n.d\right)$$

So, even if the Stacking model is very accurate, it has a noticeable computational cost. However, the meta‐learner (LR) balances overfitting and keeps the final layer efficient.

#### 3.4.3 Space Complexity

The space complexity of the algorithm is due to the training data required for building our models, and will be stored within it.

##### 3.4.3.1 Base Learners

All base learners store the training data along with any intermediate results. KNN needs to store the whole training set with O(*n*⋅*d*) space only for such models. Models like SVM, NB and DTs require space proportional to the training set size and model parameters.

##### 3.4.3.2 Meta‐Learner (LR)

The space complexity is O(*d*), where d represents the number of features output by base learners. As a result, the total space complexity will be O(*n*⋅*d*) since each learner needs to allocate memory for both the training data and models.

## 4 Results and Discussion

We present the results and evaluation of our framework of ‘Stacking Ensemble Model’ (SEM) approach. We used various performance metrics such as precision, accuracy, F1‐score, recall (sensitivity) and ROC‐AUC to evaluate the performance of the model. All these metrics were used to evaluate the classifying ability of the model. To highlight clearly the better performance of our recommended SEM, we also tested and compared its performance with several state‐of‐the‐art baseline classifiers. The comparative analysis shows that our model achieves the best performance on all the crucial evaluation metrics. Other than the performance evaluation, we also focused on the interpretability of the proposed model. The framework is based on stacking but retains the ability to reflect the workings of individual classifiers, thereby combining the strengths of multiple classifiers.

### 4.1 System Configuration

The SEM used in this study is developed in Google Colaboratory (Colab), a free cloud‐based development environment that allows writing and executing Python code in a web browser. The integration of free resources and hardware, such as CPUs and GPUs, makes Colab perfect for running ML experiments without local hardware infrastructure. In addition to the framework that builds Jupyter Notebook, the platform offers inline code creation, documentation and real‐time data visualization, which can greatly aid in creating a reproducible workflow during the development stage. The model was implemented using scikit‐learn [32], a popular Python package that has a wide array of ML techniques available. In this paper, DT, SVM and NB were used as the base learners in the ensemble framework. This setup facilitated efficient experimentation, establishing the basis for systematic comparisons of ensemble predictive performance against individual classifiers.

### 4.2 Performance Matrix

We utilized multiple performance metrics to assess the effectiveness of the ML models in detecting HD: precision, recall, F1 score and accuracy. These provide a better insight regarding the predictive capabilities of the model, especially when it comes to generalization to real world clinical data. Each of them is calculated from the confusion matrix, which summarizes the classification results of a model by comparing predicted labels with actual values. It is a matrix that can be used as a basic tool for evaluation of models from several aspects [32].

F1 score is one such informative metric because it is the harmonic mean of precision and recall. In this way, it provides an aggregated metric that discourages large differences between the two, thus favouring models that are consistently good at both maximizing true positives and minimizing false positives. Such analysis is a critical part of medical diagnostics, where a misclassification can be expensive. The evaluation metrics were calculated according to the usual formulae:

Accuracy:

$$\mathrm{ACC}\;=\frac{ɡɡ+ɡɡ}{ɡɡ+ɡɡ+ɡɡ+ɡɡ}$$

Precision

$$\mathrm{Precision}=\frac{ɡɡ}{ɡɡ+ɡɡ}$$

Recall

$$\mathrm{Recall}\;=\frac{ɡɡ}{ɡɡ+ɡɡ}$$

F1‐Score

$$F-\mathrm{Score}=2\times \frac{\mathrm{Precision}\times \mathrm{Recall}}{\mathrm{Precision}+\mathrm{Recall}}$$

### 4.3 Analysis of Findings

The training and testing accuracy of different ML classifiers is demonstrated in Table 3. This emphasizes the amazing performance of the proposed SEM. The stacking model achieves a 99.894% training accuracy and a 93.697% testing accuracy, which are significantly higher generalization numbers than any of the individual models. The high accuracy on the test data demonstrates that the stacking model was able to effectively learn from the training dataset and generalize to new and unseen data. Stacking achieves this using a meta‐learner that learns to maximize the output of multiple base learners, such as DT, KNN, and NB, while creating new features. It thereby improves overall prediction ability by reducing particular model drawbacks. On the other hand, Individual models like NB and LR suffer from underfitting or simplifying assumptions, reducing testing performance. Consequently, the stacking model is the most efficient and trustworthy of all the evaluated classifiers. Comparatively, the DT classifier performs significantly, achieving 86.554% in testing, while achieving 100% training accuracy, indicating overfitting, where the model learns from the training data but fails to generalize. Other models, like KNN (85.714% testing), NB (83.193%), Multi‐Layer Perceptron (88.235%) and LR (84.714%), exhibit either lower accuracy or greater gaps between their training and testing accuracies.

**TABLE 3:** Training and testing accuracy of the SEM.

Classifier name	Training accuracy	Testing accuracy
**Proposed Stacking Model**	**99.894%**	**93.697%**
DT	100%	86.554%
KNN	90.126%	85.714%
NB	84.033%	83.193%
Multi‐layer perceptron	94.012%	88.235%
LR	82.037%	84.714%

*Note*: Bold values indicate the performance of the proposed method.

Table 4 compares the performance metrics of the proposed SEM with those of several widely used ML classifiers. The table includes key evaluation criteria such as precision, AUC, recall, MAE, F1‐Score, MSE and RMSE, enabling a clear and detailed assessment of each model's effectiveness.

**TABLE 4:** A comparison between the SEM and other ML models.

Classifier name	Precision	Recall	F1‐score	AUC	MAE	MSE	RMSE
**Proposed Stacking Model**	**0.930**	**0.930**	**0.930**	**0.947**	**0.067**	**0.067**	**0.259**
DT	0.870	0.870	0.870	0.901	0.134	0.134	0.367
KNN	0.860	0.860	0.860	0.911	0.143	0.143	0.378
NB	0.840	0.830	0.830	0.898	0.168	0.168	0.409
Multi‐layer perceptron	0.880	0.880	0.880	0.933	0.118	0.118	0.343
LR	0.860	0.860	0.860	0.897	0.142	0.142	0.377

*Note*: Bold values indicate the performance of the proposed method.

We used identical data for each classifier in our evaluation. The SEM has 0.86 accuracy, 0.88 recall and 0.85 F1‐score. Our algorithm has the highest AUC of 0.73 among the three methods. Our model has high accuracy (93.69%), which might help to reduce the risk of misdiagnosis and thus make diagnosis more reliable in HD detection. This could provide more confident diagnosis in a clinical environment, and help to identify at risk patients and implement targeted interventions early to prepare for treatment. The high sensitivity of the model minimizes false negatives, which is crucial for early diagnosis. For HD, better recognition is particularly vital; it can be a matter of life and death.

By providing a treatment pathway earlier in HD that might prevent downstream complications, this model may assist a small proportion of people with HD in avoiding a diagnosis. This could result in very high false positive rates (FPR) with HD prediction, resulting in unwarranted further testing and procedures, causing unnecessary, perhaps excessive, anxiety to the patient and increased healthcare costs. From a clinical perspective, false negatives, which indicate missed diagnoses, are even more problematic.

In the end, failing to diagnose HD could delay the timely treatment required to deal with a serious condition, which has been linked to negative health outcomes. However, we argue that/sensitivity is the most important goal to avoid missing clinically relevant findings (false negatives), which might influence patients’ safety. The F1‐score, which combines both precision and recall, is a useful metric. In healthcare, it is important to find a balance between false positive and false negative rates. We have done a detailed analysis of the high F1‐score of our model, which justifies the robustness in reducing both types of errors. We also discuss the clinical utility of using model predictions to inform decision‐making.

**FIGURE 4:**
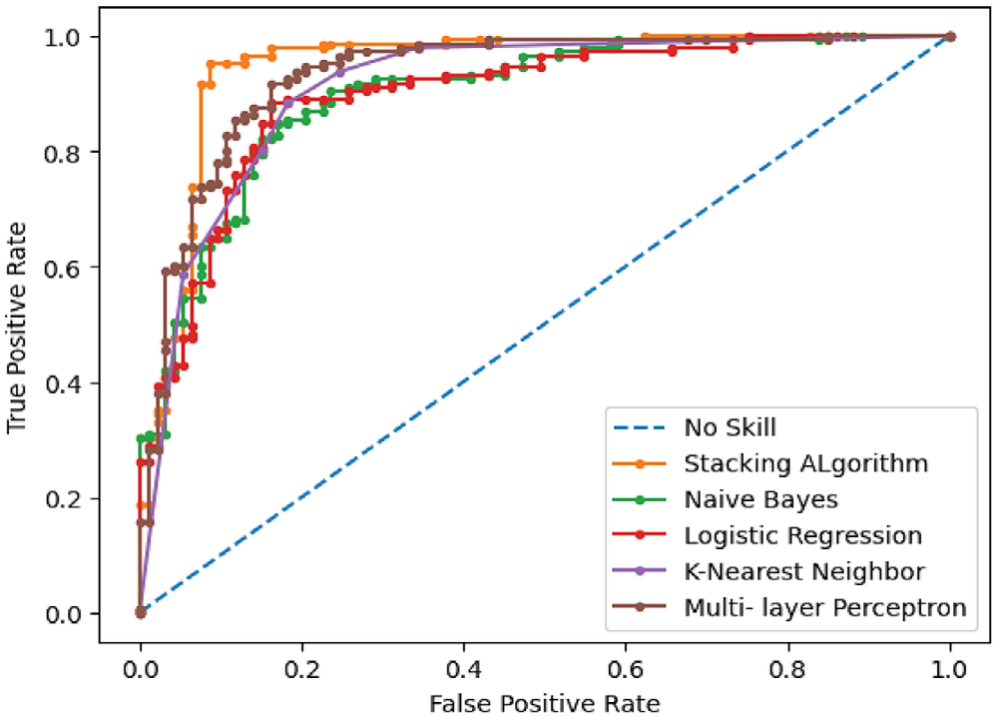
ROC curve.

Figure 4 shows the ROC curves for different ML models employed for HD prediction. It shows the True Positive Rate (TRP) against the FRP to compare the effectiveness of each model in separating the positive and negative cases. Among all, SEM demonstrates better performance, as its curve is closest to the top‐left corner, indicating better sensitivity with a lower FPR. Following KNN and LR, which demonstrate only modest efficacy, the Multi‐Layer Perceptron also performs well. Naïve Bayes has less predictive power because its curve is lower than the others. The dashed diagonal line represents a no‐skill classifier, whose performance falls short of random guessing. Generally, the figure shows that across all models, the stacking model provides the most reliable and precise classification.

**FIGURE 5:**
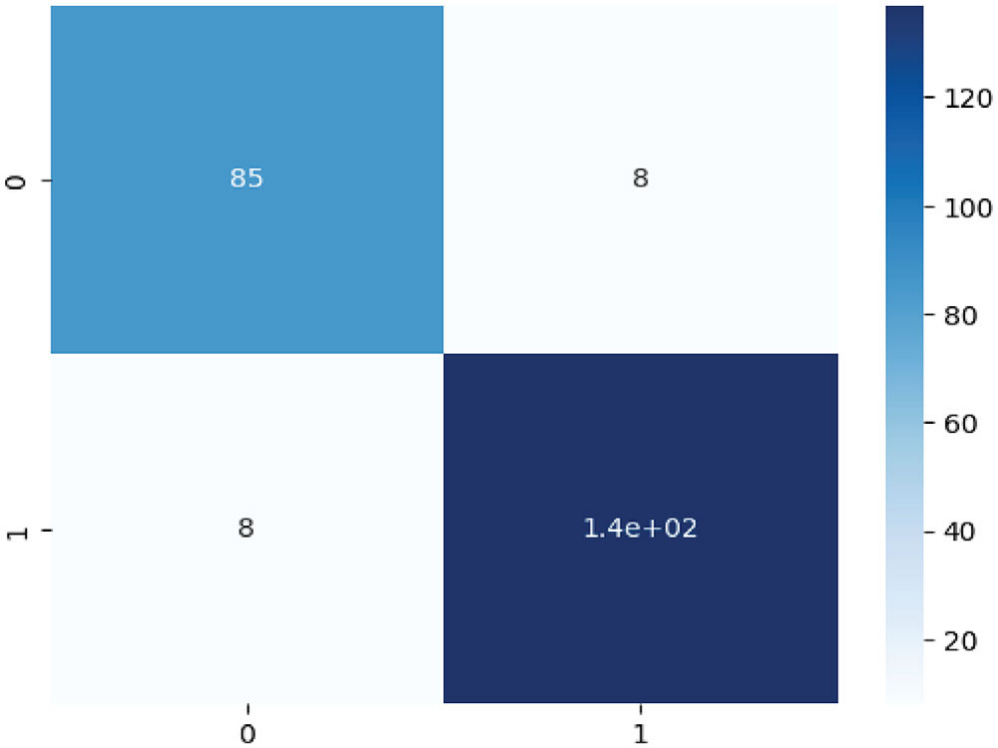
Confusion matrix of the stacking ensemble method.

The confusion matrix illustrated in Figure 5 was used to evaluate the classification performance of the HD prediction model. It visually represents the number of predictions made correctly and incorrectly by the model.

Table 5 summarizes and compares recent studies on HD prediction using various datasets and ML methods. It highlights the performance of each approach based on accuracy and F1‐score. Among the reviewed works, models such as stacking with 10 base learners, QML, CART and deep learning with feature augmentation achieved competitive results, with accuracies ranging from 84% to 90%. However, the proposed two‐stage stacking algorithm in this study outperforms all previous methods, achieving the highest accuracy of 93.69% and an F1‐score of 93% on the Heart Disease Comprehensive Dataset. This demonstrates the effectiveness and reliability of the proposed model in accurately predicting HD compared to existing techniques.

**TABLE 5:** Comparison of the stacking ensemble method with existing work.

Author	Dataset	Method	Performance matrices
Liu et al. [15]	Heart disease comprehensive dataset	Stacking (10 base learners)	ACC = 89.86%, F1‐score = 91.35%
	Heart attack dataset	Stacking (10 base learners)	ACC = 84.62%, F1‐score = 86%.
Abdulsalam et al. [24]	Cleveland benchmark dataset	Ensemble quantum ML (bagging QSVC)	ACC = 90.16%
Kumar et al. [33]	Cleveland benchmark dataset	Quantum random forest	ACC = 89%, F1‐score = 88%
Diwan et al. [34]	Heart disease comprehensive dataset	CART	ACC = 87%, F1‐score = 87.24%
Latha et al. [7]	Cleveland benchmark dataset	The majority voted with NB, BN, RF and MP	ACC = 85.48%
García‐Ordás et al. [35]	Heart disease comprehensive dataset	Deep learning method with feature augmentation	ACC = 90.088%
Proposed method	Heart disease comprehensive dataset	Two‐stage stacking algorithm	ACC = 93.69%, F1‐score = 93%

### 4.4 Model Explainability

There are two main levels of explainability: local explainability and global explainability. Global explainability makes it possible to explain the final decision at every level of every data point. It gives a quick assessment based on global fidelity. It basically specified the importance of the instance level [36]. Local fidelity could explain all the samples. It offers a clearer explanation. We use both dataset‐level global explainability and instance‐level local explainability to examine the output's final decision, identifying the causes and describing the best model (Stacking ML).

#### 4.4.1 Global Explainability

**FIGURE 6:**
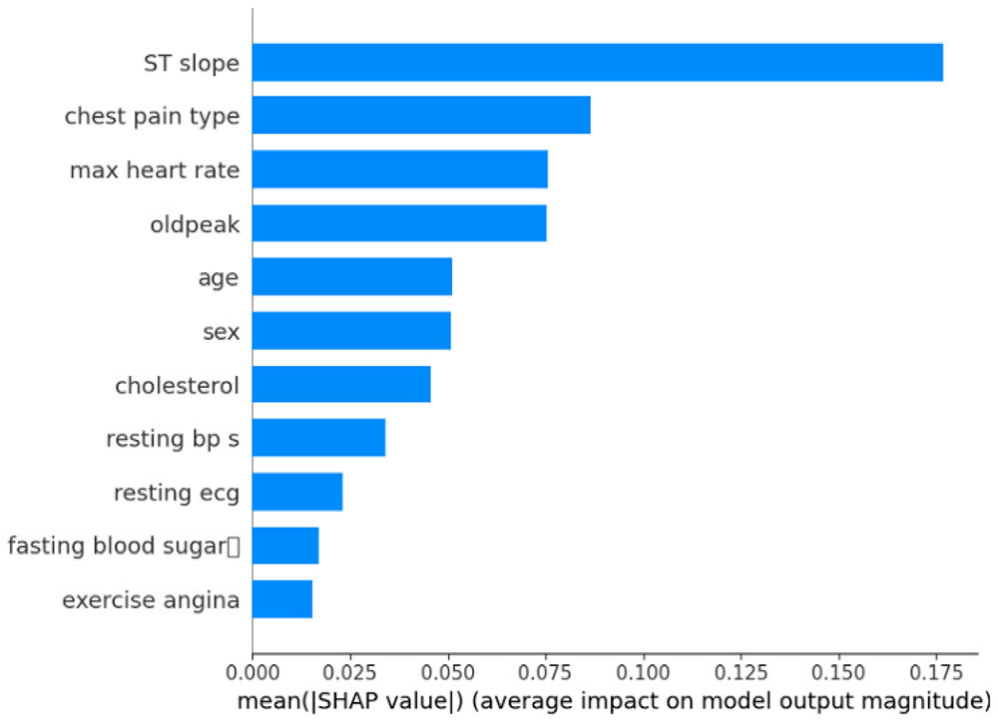
Global feature contribution.

Figure 6 shows a bar plot that shows how much each input feature affects the model's predictions. This visualization shows both high‐impact and lower‐weighted features, revealing an entire view of how each variable affects the model's output. We used SHAP values to measure feature importance. We averaged the values across all instances in the dataset to make a ranked list of contributions. ‘ST slope’ is the most important feature, followed by chest pain type, then ‘max heart rate,’ last, ‘oldpeak’. This indicates that these factors impact the model output significantly. ‘Age,’ ‘sex’ and ‘cholesterol’ are middle‐ground affecters ‘resting BP S’ and ‘resting ECG’ not so much. The ‘fasting blood sugar’ and ‘exercise angina’ are the two features which have minimal impact on prediction as they only make a minor contribution. This image visualizes the importance of each variable to the decisions made by the model, from most important to least.

**FIGURE 7:**
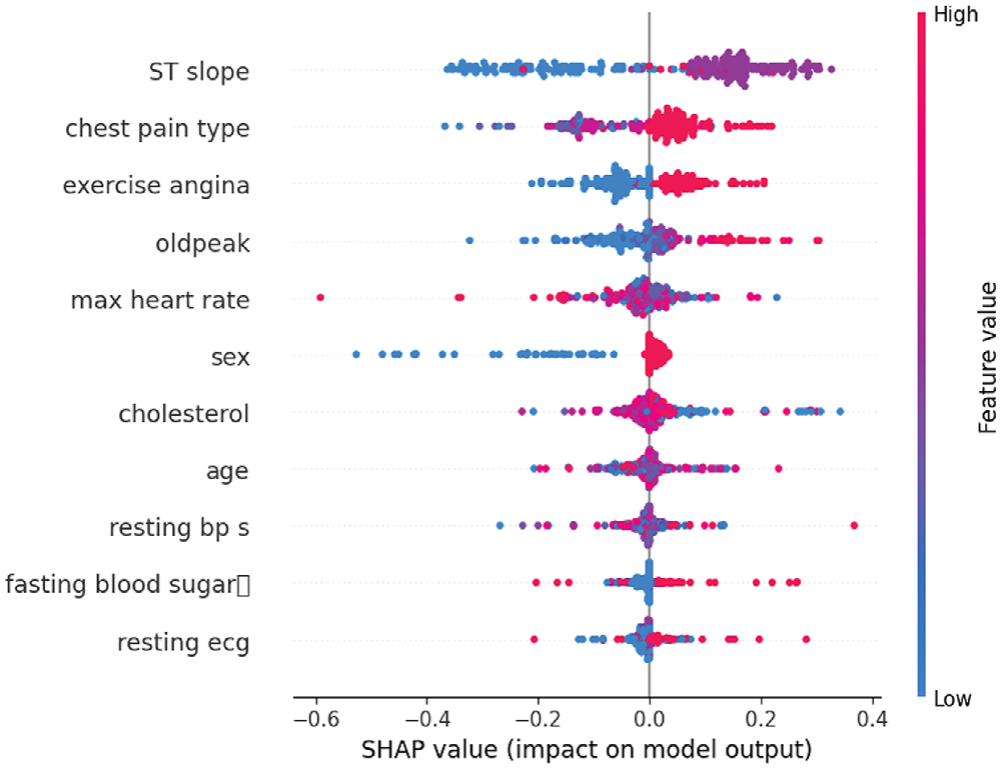
SHAP summary plot.

A SHAP summary plot is shown in Figure 7, which provides a global view of how the value of each feature impacts the model prediction for all the samples. This plot contains two key pieces of information: the colour of each individual point and their distance from the origin along the horizontal. Higher values are depicted in red and lower values are depicted in blue. Each of the feature values are represented by colour. The *x*‐axis position of each point indicates the direction and magnitude of the feature's impact on the model output. As it is a binary classification problem for the model, the output is a score between 0 and 1 representing class probabilities. If SHAP values for the features extend to the right (positive side), the probability of a positive class prediction increases, and if the feature goes to the left (negative side) the probability decreases. Features are ranked vertically in order of average whole SHAP value across the dataset. It gives a ranking of the predictors in order of how much they affect the decisions of the model. Particularly, ST slope and chest pain type had the largest overall effects indicating that they are clinically significant regarding predicting HD. These findings align with well‐established pathophysiological patterns, for example, ST‐segment changes and specific attributes of chest pain are classical cardiovascular signs.

**FIGURE 8:**
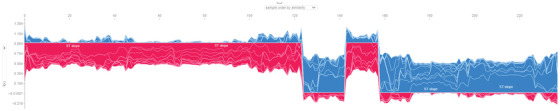
Stacked SHAP force plot.

A stacked SHAP force plot is a visualization that combines a number of force graphs that display the prediction when observing every case in the dataset. This is a single plot showing the predictions for your samples, the same way we created the stacked plot. This is achieved by rotating the force plots of all instances by 90° and stacking them vertically according to their clustering similarity. The *x*‐axis represents individual data instances, while the *y*‐axis corresponds to the baseline prediction. Figure 8 displays the stacked SHAP force plot of the model. Greater values on the vertical axis indicate an increased risk of HD, whereas lower values indicate a decreased probability of HD. The red features enhance the model score, elevating it to the highest level, whereas the blue features decrease the score, causing it to decline to the lowest level.

**FIGURE 9:**
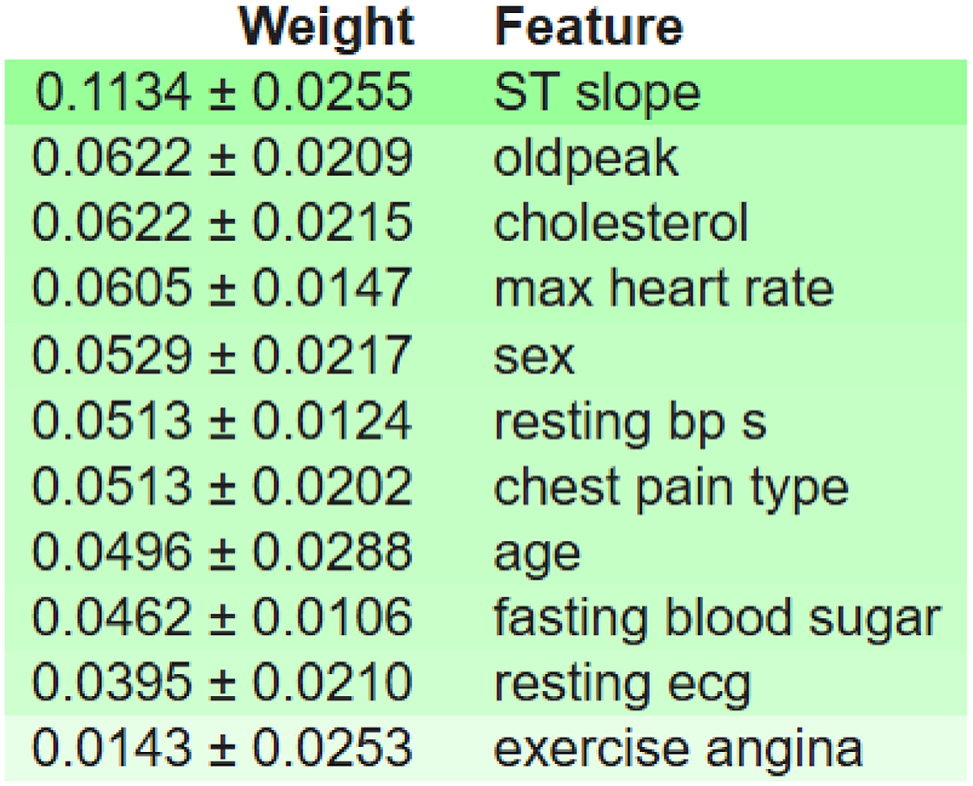
Permutation importance plot.

Figure 9 is an ELI5 permutation‐importance table, where each feature is randomly shuffled to recalculate the model's performance. The Weight indicates the average drop in the selected score (accuracy/AUC, for example) brought on by the shuffling, meaning that larger is more significant. The standard deviation of the drop across repeats/CV folds, or the estimate's uncertainty, is represented by the ‘±’ value. The most important factor in this case is the ST slope (0.1134 ± 0.0255); if it is scrambled, it will negatively impact the performance the most. Old peak, cholesterol, maximum heart rate, sex, resting systolic blood pressure, type of chest pain, age, fasting blood sugar, resting ECG and exercise angina have the least impact. Correlations between features may mitigate interpretations, which are specific to the metric and model (importance can be shared or swapped among correlated variables). Features are ranked overall according to their importance for the predictions made by the trained model, with uncertainty displayed for each estimate.

#### 4.4.2 Local Explainability

Local explainability focuses on figuring out why a certain outcome happened by figuring out which features had the greatest effect on it. This kind of explanation is particularly needed when it's more important to know why a certain choice was made than to figure out how the model works. By applying local explainability methods to our HD prediction model, we gained insights into individual patients' decision‐making. SHAP was applied to estimate the contribution of each feature to the difference between the prediction by the model and a baseline value. It provided a relatively simple justification for each one. In addition, we utilized LIME, which employs a simple, interpretable model to predict the model's assumptions in a local region. These techniques allowed us to validate and interpret predictions made by our models, which in turn increased confidence in the model's ability to assist in the diagnosis of HD.

The SHAP force plot is an essential technique for local explanation that allows us to appreciate the influence of each feature on the output and how every model is affected. It demonstrates the effect of each feature on the prediction, how it has changed the prediction compared to a baseline value. The red arrows indicate features that contribute to increasing the prediction, while the blue arrows indicate features which contribute to decreasing it. The size of each arrow represents the strength of the effect. It helps us understand why a model predicted some value for a particular case. In our HD prediction model, an SHAP force plot demonstrated which features most affected each patient's risk prediction. This made the model's output easier to understand and more reliable.

**FIGURE 10:**
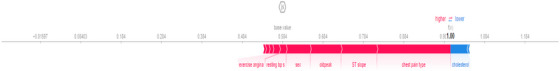
SHAP force plot for Instance 1.

Figure 10 is a SHAP force plot, which visualizes how every feature impacts the prediction of a specific instance. The baseline value of 0.594 represents the model's average output for all the data. In this case, the final model prediction reached 1.00, indicating a strong prediction that the positive class (probably HD presence) would be the case. The model was more likely to predict HD because of features in red, like exercise angina, resting blood pressure, sex, oldpeak, ST slope and chest pain type. On the other hand, the feature cholesterol, which is shown in blue, had a harmful effect and slightly lowered the prediction value. The length and colour of each arrow show how strong that feature's effect is. In general, this SHAP plot shows that the model's decision in this case was heavily influenced by the combination of several risk‐related features.

**FIGURE 11:**
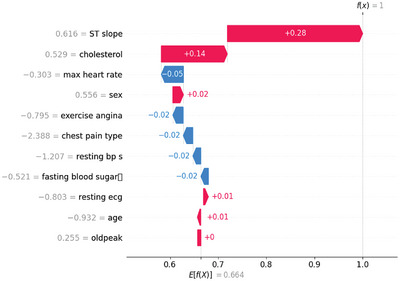
SHAP waterfall for Instance 9.

Figure 11 shows the SHAP waterfall, which shows why Instance #9 is predicted to be Class 1 with high confidence. The model begins with the dataset baseline E[f(*X*)] = 0.664 and, through feature contributions, approaches f(*x*) = 1.0. The score goes up when the bars are red and down when they are blue. The most significant increases are in ST slope (+0.28) and cholesterol (+0.14). Sex and resting ECG add a little more (+0.02 each), while oldpeak has no effect. The prediction is lower because of chest pain type (−0.05), maximum heart rate (−0.02) and age (−0.02). There are also minor negatives from exercise angina, fasting blood sugar and resting BP (each about –0.01 to –0.02). Overall, the strong positives, especially the ST slope and cholesterol, more than make up for the minor negatives, which brings the final prediction close to 1.

**FIGURE 12:**
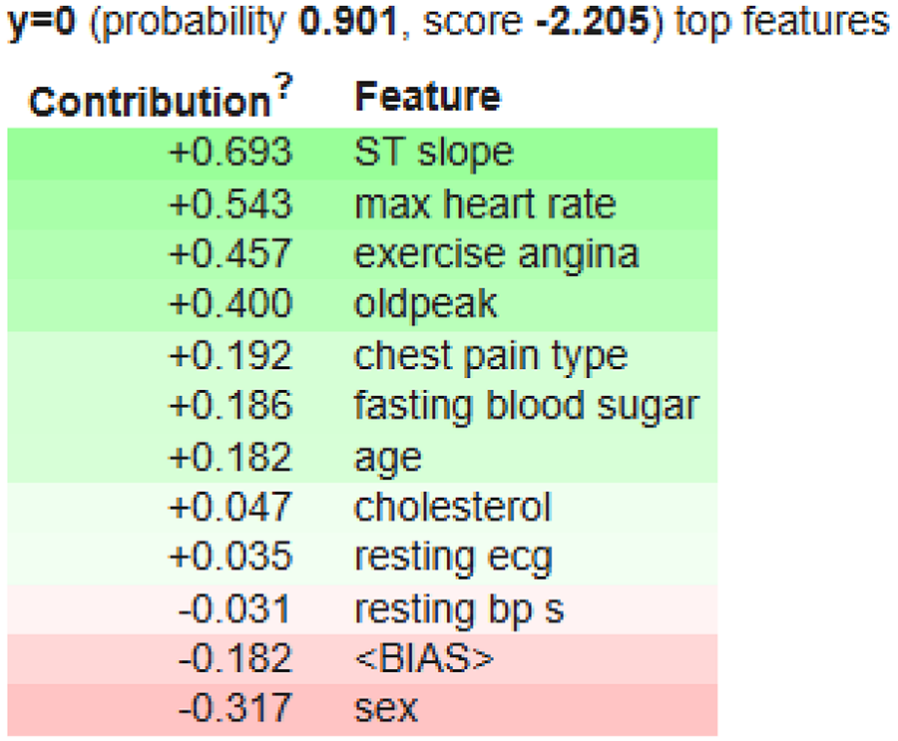
Eli5 panel for Instance 10.

Figure 12 shows that the ELI5 panel explains why Instance #10 is predicted to be Class *y* = 0 with a 90.1% chance. The ‘score’ is the raw log‐odds, which in this case are –2.205 for *y* = 1 and +2.205 for *y* = 0. The ‘contributions’ of the listed features add up to that value. The red rows tend to the prediction toward *y* = 1, while the green rows tend to it toward *y* = 0. The most convincing evidence for *y* = 0 comes from ST slope (+0.693), max heart rate (+0.543), exercise angina (+0.457) and oldpeak (+0.400). There is also a minor supporting impact from chest pain type (+0.192), fasting blood sugar (+0.186), age (+0.182), cholesterol (+0.047) and resting ECG (+0.035). Sex (−0.317) and resting BP (−0.031) go against the *y* = 0 prediction, as does the model's (−0.182). However, these negatives are too small to cancel out the strong positives. The total contributions favour *y* = 0 by about +2.205 log‐odds, which means that Class 0 has a 0.901 chance of happening.

**FIGURE 13:**
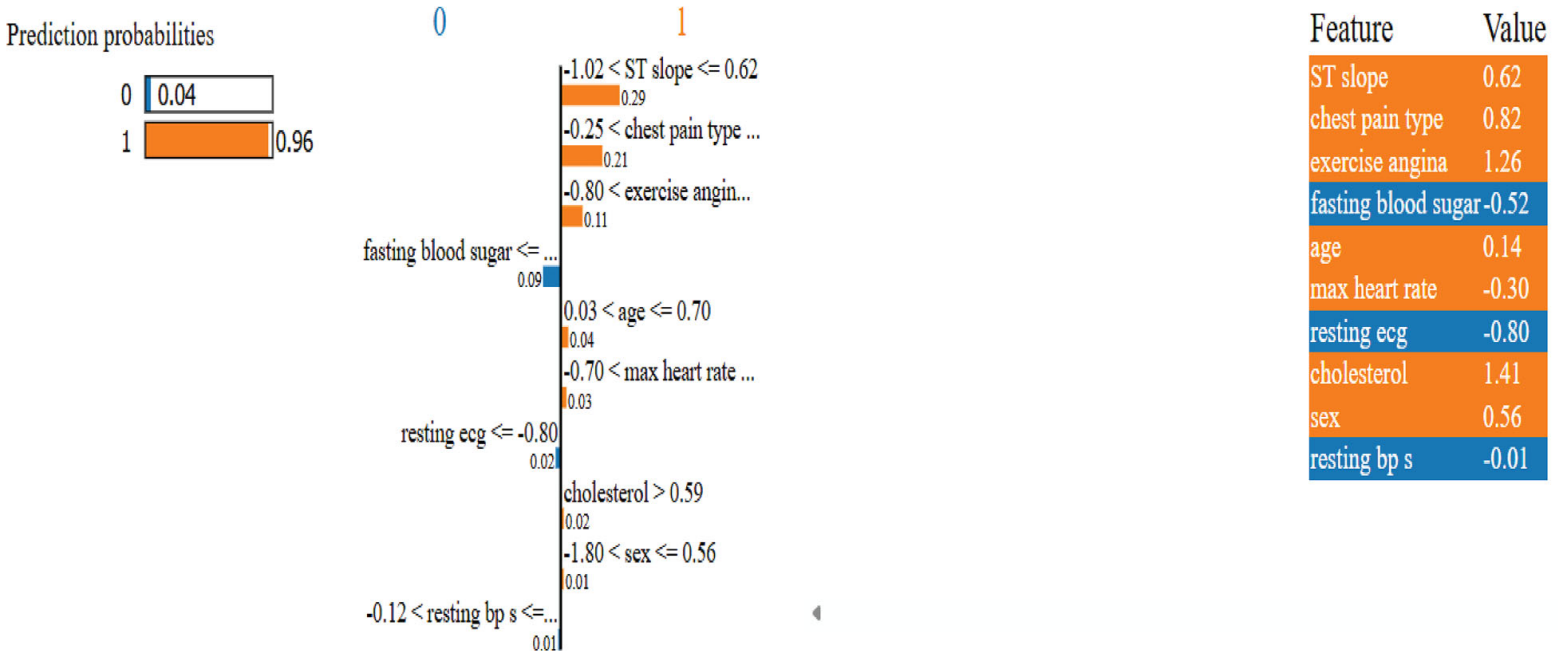
LIME explanation of the stacking model.

Figure 13 presents a LIME plot, which explains the prediction of an HD model for a specific individual instance. The prediction probability is shown on the left, where the model predicts Class 1 (presence of HD) with 100% confidence. The central bar chart displays the local decision rules (feature thresholds) and their corresponding contributions toward the prediction. Features pushing the prediction toward Class 1 (positive case) are shown in orange, while those pushing toward Class 0 (negative case) are in blue. There is a table on the right with the actual feature values of the person. Top contributors to the model output for HD are ST slope (0.62), chest pain type (0.82), oldpeak (0.26) and exercise induced angina (1.26). Conversely, cholesterol (1.41) and fasting blood sugar (−0.52) have a lower impact or a weak negative effect on the prediction. This visualization allows us to see what influences the model's decision and provides transparency to the interpretability of predictions at the individual level.

#### 4.4.3 Feature Interaction

Feature interaction occurs when two features work together in such a way that the effect of one depends on the level of the other. This is something that simple ‘one‐feature‐at‐a‐time’ views can miss. To effectively visualize ‘it‐depends’ behaviour, employ pairwise effect plots such as 2D accumulated local effects (ALE), which circumvent the deceptive averaging of partial dependence in the presence of correlated features. SHAP interaction values break down a single prediction into main effects and pairwise interaction terms, showing which pairs of features were most important for case‐by‐case explanations. Interactions help build trust and fix bugs because they reveal subgroups where the model acts differently than the global averages suggest it should.

##### 4.4.3.1 SHAP Interaction Values

**FIGURE 14:**
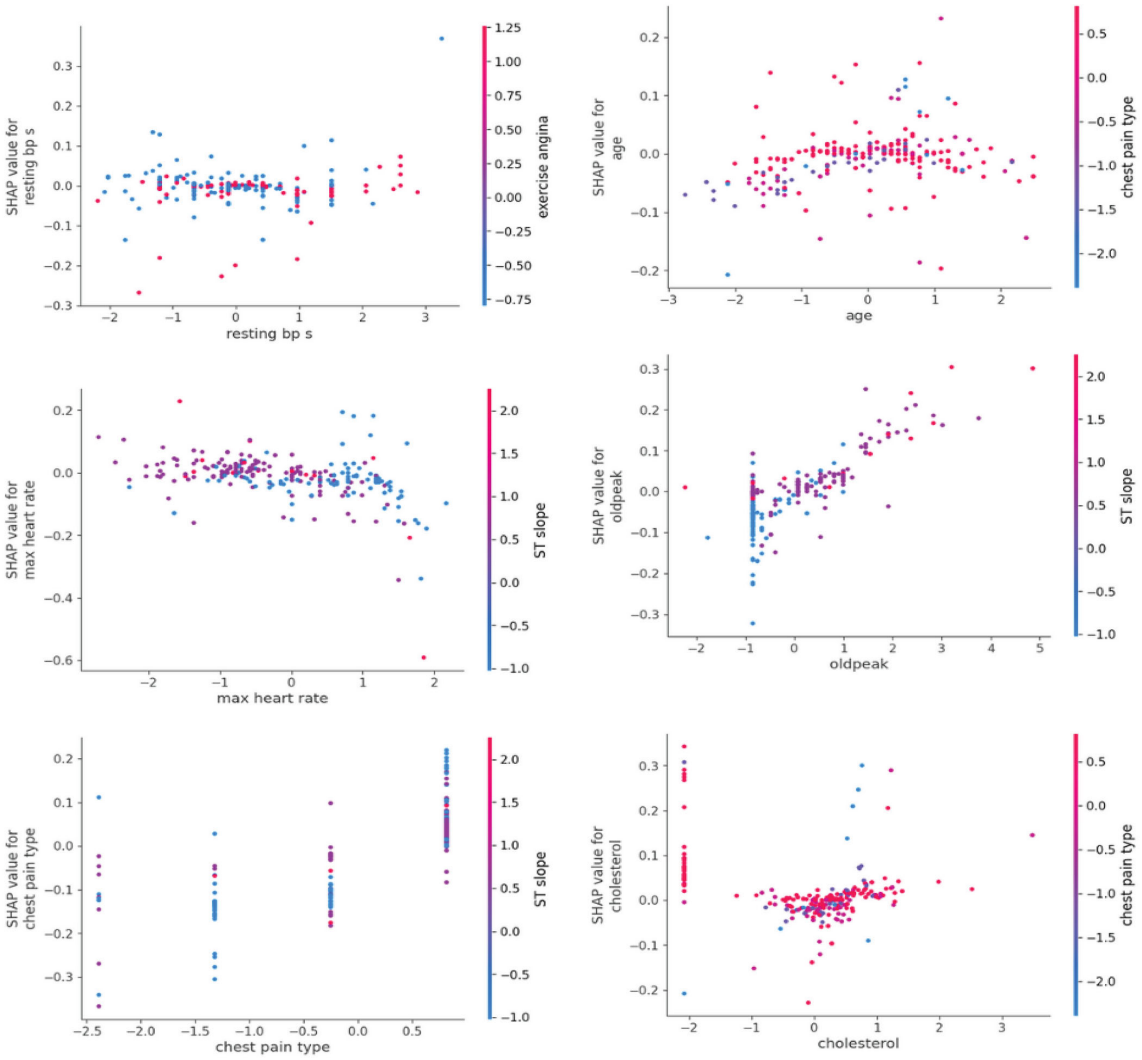
SHAP interaction values.

Figure 14 shows SHAP dependence/interaction plots. The *x*‐axis in each small panel shows the value of a feature, the *y*‐axis shows the SHAP value of that feature (how much it pushes the prediction), and the colour shows the other feature that SHAP found to interact with it the most. When points of different colours are at very different *y*‐levels for the same *x*, the two features interact (the effect of one depends on the other). The most obvious example is the ST slope, which serves as the colouring partner for many panels and has high values that cluster with larger |SHAP|. This means that ST slope changes how variables like maximum heart rate, age, oldpeak and chest pain type affect the outcome. Vertical stripes indicate categorical features, such as sex, chest pain type and resting ECG. Inside each stripe, a trend of colours up/down indicates how the colouring feature combines with others. In contrast, panels such as resting BP and cholesterol exhibit tighter clouds with more colours mixed in, indicating weaker interactions. As can be seen from the figure, the risk estimation by the model is not a simple mean across all features. For example, high ST slope and certain types of chest pain (and specific ranges of max heart rate or oldpeak) make contributions that are very different from any one feature.

##### 4.4.3.2 Partial Dependence Plot

The PDP shows how one or two features affect the predicted outcome of an ML model. We can use a PDP to determine if the connection between the target and a feature is linear, monotonic or more complicated.

##### 4.4.3.3 1D Partial Dependence Plot

**FIGURE 15:**
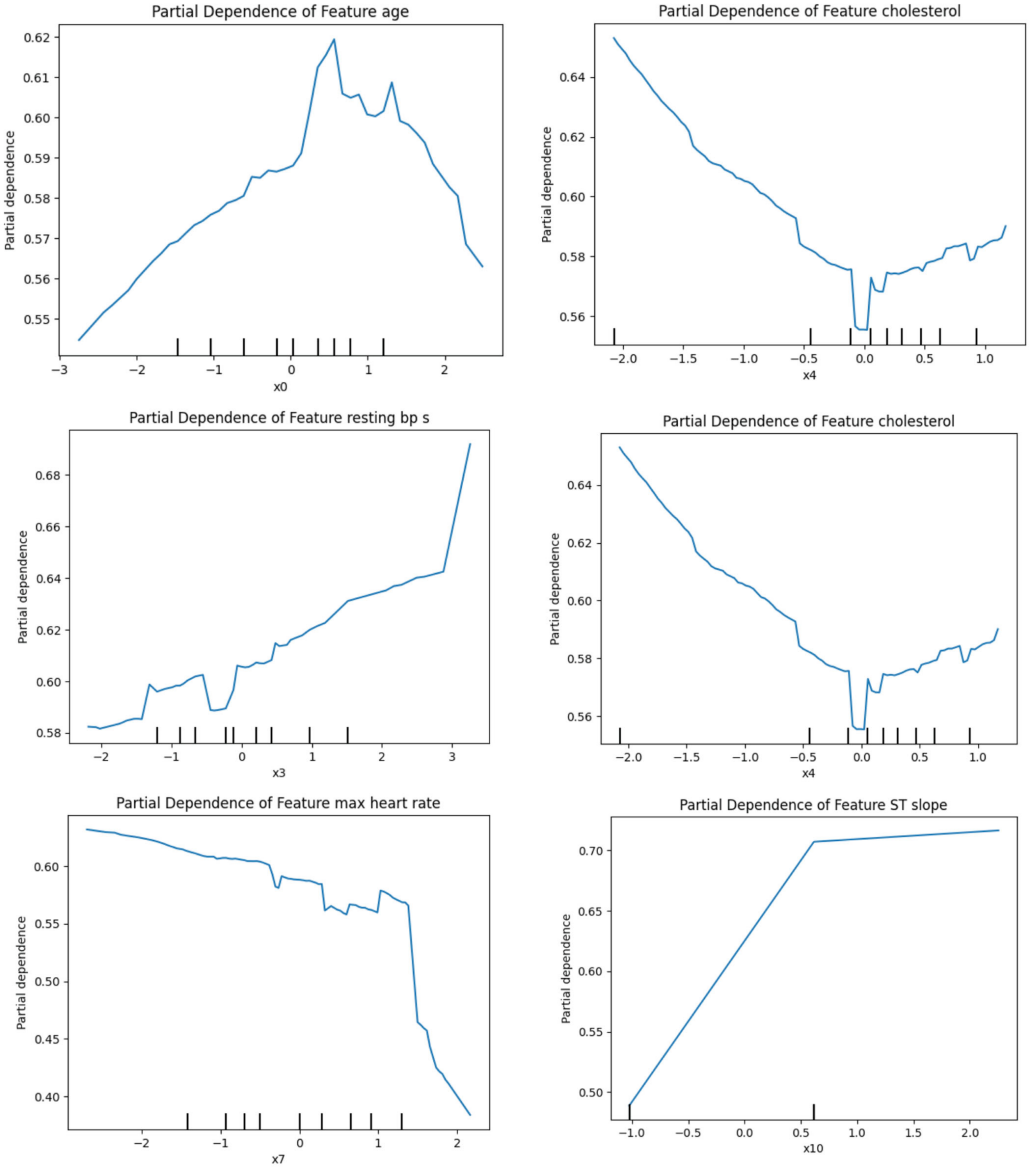
Partial dependence plot (1D).

Figure 15 is a 1D partial‐dependence plot showing how each feature affects the model on average, while keeping the others constant. The ST slope goes almost straight up, so higher ST‐slope values always mean more risk. The old peak is relatively flat at low values, but it starts to increase after about 1, which means that ST‐depression becomes significant as it becomes clearer. Overall, the maximum heart rate goes down, with a substantial drop at its highest value. This means that people who can reach a high maximum heart rate look safer than the model (there's a threshold effect). Age isn't a straight line; the risk rises with age, peaks in the middle of the range shown, and then goes down a little. This ‘bump’ shows that age's effect depends on other factors. Cholesterol goes down and then up a little, which suggests that there are groups of people with high cholesterol and other traits that change their risk. Resting systolic BP stays almost the same until it gets very high, when the risk goes up a lot. So it only matters at the ends. When it comes to interactions, bends, kinks and cliffs usually mean ‘it depends.’ Age times maximum heart rate (older + low max HR = higher risk), ST slope times oldpeak (both high = much higher risk), and cholesterol times ST slope (cholesterol's effect is stronger when ST findings are abnormal).

##### 4.4.3.4 2D Partial Dependence Plot

**FIGURE 16:**
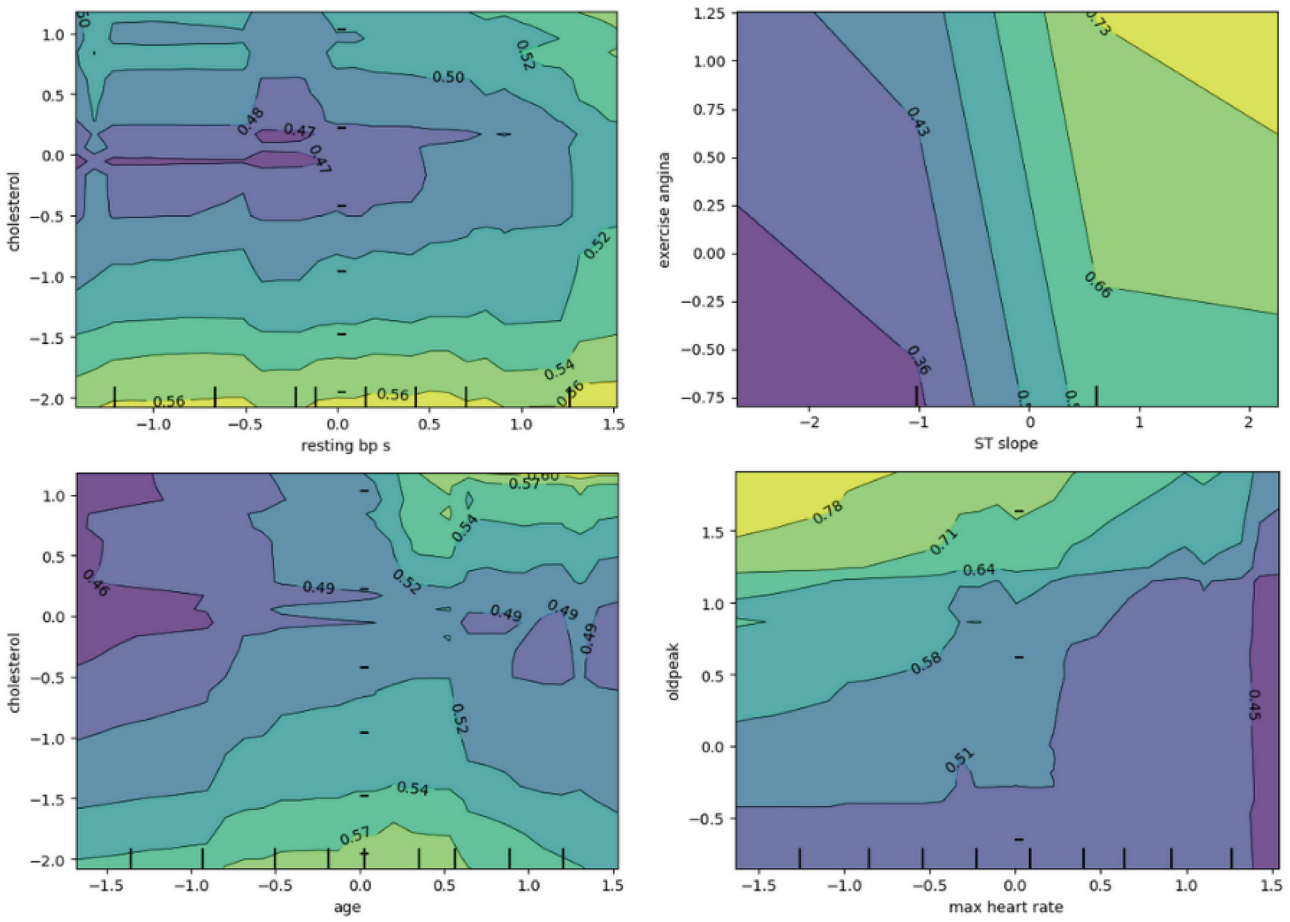
Partial dependence plot (2D).

Figure 16 is a 2D partial‐dependence contour plot. The colours and contours show the model's average predicted risk as each pair of features changes, and the tick marks on the axes show where the data is most concentrated. When both resting BP and cholesterol are low, the risk is lowest. When either one rises, the risk is higher. There is a mild interaction ridge at high BP, even with moderate cholesterol. The ST slope and exercise angina have diagonally tilted contours, which means they exacerbate each other. The most dangerous situation occurs when both are high or present. The relationship between age and cholesterol is not linear; risk usually goes up with age and cholesterol, but there are some small neutral areas (probably areas with little data). Maximum heart rate (oldpeak) shows a strong trade‐off: risk goes up with oldpeak but down with maximum heart rate. The worst areas are low max‐HR and high oldpeak. Fewer rug ticks mean fewer training samples and less reliable contours there.

#### 4.4.4 Counterfactual Explanations

In XAI, counterfactual explanations refer to the smallest changes that could be made to a case that would change a model's decision. For example, ‘what needs to change for a different outcome?’ These explanations are suggested as a useful, model‐agnostic way to help people understand, contest, and improve automated decisions, such as in GDPR contexts. Instead of just listing important features, they are useful because they turn vague predictions into specific actions that a person could take. Modern toolkits, like DiCE, generate numerous counterfactuals so that users can see many realistic ways to make a better choice. Researchers also warn that counterfactuals should follow causal and feasibility rules (some things can't be changed directly), so recourse should be viewed as possible changes, not random input flips. For example, if a model says ‘high risk’ for HD, a counterfactual could say, ‘If ST‐segment depression (oldpeak) dropped from 1.8 to 0.4 and LDL cholesterol dropped by about 30 mg/dL (with other factors held constant), the prediction would change to ’low risk.‘’ This would give doctors and patients specific goals to discuss (if they are medically possible).

**FIGURE 17:**
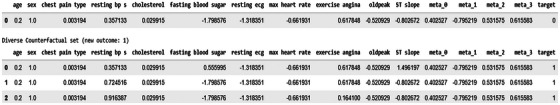
Counterfactual explanation illustrating how small variations in key features lead to a change in heart disease prediction outcome, specifically showing a shift from negative prediction values to positive prediction values.

Figure 17 provides a counterfactual explanation for a patient initially classified as Class 0 (low risk). The ‘Diverse Counterfactual set (new outcome: 1)’ shows three small changes that change the label to Class 1: (i) Age increases from 0.2 to 0.6 SD; (ii) resting systolic BP rises from 0.357 to 0.994 SD; (iii) resting BP rises to 0.827 SD and max heart rate jumps from –0.662 to 3.07 SD. All other variables, such as sex, type of chest pain, fasting blood sugar, resting ECG, oldpeak, ST slope and the meta_* features, are kept the same. This shows that there are many ways to make a different decision with only a small change. These counterfactuals are helpful for checking and figuring out how models work, but keep in mind that some changes (such as aging or increasing blood pressure) can't be acted upon or are not clinically appropriate.

**FIGURE 18:**
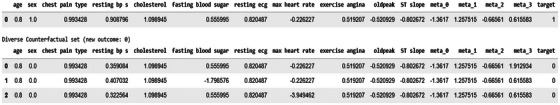
Counterfactual explanation illustrating how small variations in key features lead to a change in heart disease prediction outcome, specifically showing a shift from positive prediction values to negative prediction values.

Figure 18 shows a counterfactual explanation for a patient who was predicted to be Class 1 (high risk). The ‘Diverse Counterfactual set (new outcome: 0)’ shows three versions that would change the decision to Class 0 (low risk): (CF#1) changing sex from 1 to 0 and lowering resting systolic BP from about 0.91 to about 0.36; (CF#2) changing sex from 1 to 0 and having a noticeable drop in fasting blood sugar from 0.56 to –1.80 and a small drop in BP; (CF#3) changing sex from 1 to 0, lowering BP from about 0.32 to about 0.32, and having a huge drop in max heart rate from –0.23 to –3.95. The majority of other features (chest pain type, cholesterol, resting ECG, oldpeak, ST slope) remain unchanged. The model provides multiple avenues to achieve a lower‐risk designation, primarily through blood pressure, glucose levels and occasionally maximum heart rate. However, alterations such as sex are not actionable; therefore, utilize these findings for auditing purposes and to concentrate on clinically viable interventions.

## 5 Conclusion and Future Work

In this work, a two‐layer stacking ensemble framework for HD prediction is proposed, consisting of several base learners, including KNN, SVM, NB, DT and LR, as the meta‐learner. The proposed model achieved a high classification accuracy of 93.69% and an AUC of 94.7%, outperforming several benchmarks. This not only showed the predictive capability of the model but also solved an important clinical AI problem about transparency in model decision making. To enhance interpretability and build trust with clinicians, we added three complementary XAI techniques: SHAP, Eli5 and LIME. SHAP and Eli5 explained the global feature importance and pointed out that both ST slope and chest pain type were the strongest predictors of HD risk. On the other hand, LIME provided patient‐level explanations, aimed at enhancing the transparency of model decisions and the clinical actionability of their outputs. These tools provide a solid interpretability layer that connects model performance to the explainability demanded by real‐world healthcare environments.

This work's primary contribution is showing that interpretability and high model accuracy are not mutually exclusive. The integration of EL techniques within interpretable frameworks is supported by this work. It also offers useful advancements in the application of ML models in clinical settings, allowing for prompt, transparent and useful predictions for cardiovascular risk assessment that may influence patient outcomes. However, several limitations remain. In this work, we trained and evaluated the model on openly available data, which, despite being more widely used to evaluate generalizability, remains limited compared to diverse clinical populations in real‐world settings. Hence, it needs to be validated on larger, more heterogeneous real‐world datasets to assess its performance and generalization. Furthermore, even though SHAP and LIME are interpretable, their use in clinical workflows requires a user‐friendly interface, along with training for healthcare professionals who are not familiar with these tools. The SEM can also be computationally complex, which can make integration in time‐critical or resource‐constrained environments difficult.

Further development can be achieved to improve the scalability of the model and its integration into a near real‐time system. To enhance robustness and contextual specificity, integration of data from electronic health records, wearable sensor data or longitudinal clinical observations could help to improve measures. Alternatively, investigating deep learning models with built‐in interpretability, such as attention mechanisms or naturally interpretable architectures, may provide deeper insights without sacrificing effectiveness. Physicians, regulatory specialists and end users must work with the technical community to develop clinically deployable systems that ensure ethical, more usable and impactful AI deployment in cardiovascular care.

## Author Contributions

**Nazmun Nahar**: conceptualization, methodology, software, formal analysis, resources, data curation, writing – original draft preparation. **Sanjatul Hasan Siam**: methodology, software, formal analysis, resources, data curation, writing – original draft preparation. **Joy Bhowmik**: methodology, software, formal analysis, resources, data curation, writing – original draft preparation. **Ayesha Nasrin Ripa**: methodology, formal analysis, data curation, writing – original draft preparation. **Md. Hasan Imam**: methodology, formal analysis, data curation, writing – original draft preparation. **Haw Jiunn Woo** and **Hamid Osman**: software, resources, visualization. **Mayeen Uddin Khandaker**: conceptualization, writing – review and editing, supervision, visualization. **Shams Forruque Ahmed**: writing – review and editing, supervision, visualization.

## Conflicts of Interest

The authors declare no conflicts of interest.

## Data Availability

Data available on request from the authors.
